# Spatially and optically tailored 3D printing for highly miniaturized and integrated microfluidics

**DOI:** 10.1038/s41467-021-25788-w

**Published:** 2021-09-17

**Authors:** Jose L. Sanchez Noriega, Nicholas A. Chartrand, Jonard Corpuz Valdoz, Collin G. Cribbs, Dallin A. Jacobs, Daniel Poulson, Matthew S. Viglione, Adam T. Woolley, Pam M. Van Ry, Kenneth A. Christensen, Gregory P. Nordin

**Affiliations:** 1 Electrical and Computer Engineering Department, Brigham Young University, Provo, UT 84602 USA; 2 Chemistry and Biochemistry Department, Brigham Young University, Provo, UT 84602 USA

**Keywords:** Lab-on-a-chip, Electrical and electronic engineering

## Abstract

Traditional 3D printing based on Digital Light Processing Stereolithography (DLP-SL) is unnecessarily limiting as applied to microfluidic device fabrication, especially for high-resolution features. This limitation is due primarily to inherent tradeoffs between layer thickness, exposure time, material strength, and optical penetration that can be impossible to satisfy for microfluidic features. We introduce a generalized 3D printing process that significantly expands the accessible spatially distributed optical dose parameter space to enable the fabrication of much higher resolution 3D components without increasing the resolution of the 3D printer. Here we demonstrate component miniaturization in conjunction with a high degree of integration, including 15 μm × 15 μm valves and a 2.2 mm × 1.1 mm 10-stage 2-fold serial diluter. These results illustrate our approach’s promise to enable highly functional and compact microfluidic devices for a wide variety of biomolecular applications.

## Abstract (web summary)

The ever-growing need for highly functional, compact, and integrated microfluidic devices often incurs lengthy and expensive manufacturing processes. Here, authors introduce a generalized 3D printing process that enables fast parallel fabrication of miniaturized, high resolution 3D components.

## Introduction

The overarching characteristic of fabricating microfluidic devices is creating negative space, i.e., voids, in an otherwise solid material^1^. Traditional methods to create such voids rely on either planar fabrication using cleanroom processes or plastic forming methods such as hot embossing or injection molding. In each case, individual layers are fabricated that must be aligned and bonded, with one or more layers having surface relief features that result in voids when stacking layers in the bonded device^2,3^. Using such processes for microfluidic device development incurs significant delays in the iterative design/fabricate/test cycle required to create successful devices, too often making device development a lengthy and expensive process. One reason 3D printing has received so much attention in recent years for microfluidic device fabrication^4–12^ is that it offers the potential to dramatically speed up device development by reducing the fabrication part of the development cycle to the minutes timescale. Moreover, 3D printing affords the additional benefit of moving away from planar or stacked designs, enabling actual 3D layout of microfluidic elements with complex yet compact 3D geometries, resulting in the fabrication of devices that contain elements not possible with conventional planar methods^13,14^.

Based on previous work^15,16^, Digital Light Processing Stereolithography (DLP-SL) offers a particularly attractive approach to 3D print microfluidic devices because at least tenfold higher resolution can be realized than with the closest competitor method, inkjet-based material jetting (compare Refs. ^16,17^), and much larger build sizes can be fabricated in much less time than with submicron resolution two-photon polymerization^18,19^. However, current commercial DLP-SL 3D printing practice uses an overly limiting process comprised of the following steps: (1) design device in 3D CAD tool, (2) export design as STL (Standard Tessellation Language) file, (3) slice STL file into a stack of 2D images in which each image represents a layer of identical thickness, (4) perform 3D printing by exposing each image to the same thickness of resin using the same exposure time for all images above the initial burn-in layers, (5) post-print flush to remove unpolymerized resin and thereby reveal the interior features (voids) that comprise the device, and (optionally) (6) post-flush cure to drive further polymerization to increase material strength.

The above process can be effective for non-microfluidic designs with positive features where the minimum feature size is 5–10 pixels (100–200 μm) or larger. However, a critical consequence of Steps 3 and 4 is that the entire 3D print represents a complex set of trade-offs involving layer thickness and exposure time that can be impossible to satisfy, especially for high-resolution microfluidic device features involving only a few pixels. For example, the need for adequate green (as-printed) material strength mandates longer layer exposure times, which is vital so that the overall printed part can be connected to a vacuum and/or pressure source to flush unpolymerized resin from void regions, and so that otherwise fragile membranes in valve structures can withstand the forces inherent in the flushing process. However, longer exposure times result in deeper optical penetration into previously fabricated layers during exposure, making it impossible to create the smallest z features because trapped unpolymerized resin in negative spaces becomes polymerized. Hence, standard DLP-SL 3D printing imposes severe fabrication limitations on the size and type of microfluidic device structures, which artificially limits the potential of 3D printing for 10–30 μm feature microfluidic device fabrication.

The purpose of this paper is to introduce transformational changes to traditional 3D printing as described above, enabling negative structures as small as a few pixels to be created, including active features such as valves. Our generalization of the 3D printing process includes the following characteristics: (1) each layer can be composed of an arbitrary number of overlapping and/or spatially distinct images such that arbitrary position-dependent optical exposure is achieved within each layer, (2) each layer can have an arbitrary thickness, independent of all other layers, and (3) multiple stacked layers of smaller thicknesses and limited spatial extent can be embedded in surrounding thicker layers. These features make it possible to mix and match arbitrary layer thicknesses and exposure regions to access a much larger x/y/z/dose photopolymerization parameter space than traditional 3D printing and thereby break its unnecessarily restrictive tradeoffs to achieve much smaller active elements for a given raw resolution of the 3D printing system. We note that our previous demonstration of 18 μm × 20 μm 3D printed passive channels made with a custom 3D printer and resin employed only Characteristic 1^16^.

Using our generalized 3D printing approach, we demonstrate dramatic miniaturization of active components. For example, we show that 3D printed membrane valves can be reduced from a membrane diameter of 40 pixels (300 μm)^20^ to 6 pixels (46 μm). In addition, we introduce a few-picoliter dead volume 3D printed valve, called a squeeze valve. We show that squeeze valves can have an active area as small as 2 × 2 pixels (15 μm × 15 μm). We use both types of valves to create compact pumps. We then integrate multiple pumps and valves into compact, fast, diffusion-driven, 1:1 mixers, and sequentially connect ten individual mixer units to create a serial dilution system with ten simultaneous discrete outputs having relative concentrations that span three orders of magnitude. When squeeze valves are used, the ten-stage serial diluter is exceptionally small, having an x–y footprint of only 2.2 mm × 1.1 mm. Finally, we illustrate the utility of an on-chip serial dilution system by demonstrating the dose-dependent permeabilization of A549 cells in different concentrations of digitonin. The microfluidic device miniaturization and integration shown in this paper demonstrate the transformational potential of our re-envisioned 3D printing approach.

## Results

### Miniaturization of 3D printed valves

On-chip integrated valves are critical components to control fluid flow in microfluidic devices and are an important element in the popularity of polydimethylsiloxane (PDMS) microfluidics in which it is straightforward to create valves based on the elastomeric nature of PDMS^21^. Therefore, there has been a strong motivation to endow 3D printed microfluidics with a similar valve fabrication capability. The first demonstration of 3D printed valves was shown in 2015 by our group^7^, closely followed by the Folch group^8^. In both cases the valves were not particularly small, 2 and 3 mm diameter and 10 mm diameter, respectively. Since then, there has been a continual drive to reduce the size of 3D printed valves. In 2016 we reported 1.08 mm diameter valves using a commercial 3D printer and custom resin^22^. With the advent of our first custom 3D printer and associated resin^16^, we showed in 2018 the realization of 300 μm diameter valves^20^. In 2019 Folch et al. demonstrated 500 μm diameter valves using a commercial 3D printer and a custom resin^11^. In this paper we use our approach to 3D printing to demonstrate 3D printed membrane valves as small as 46 μm in diameter. Moreover, we show that this 3D printing approach enables the fabrication of squeeze valves that are even smaller, down to ~15 μm × 15 μm.

**Fig. 1:**
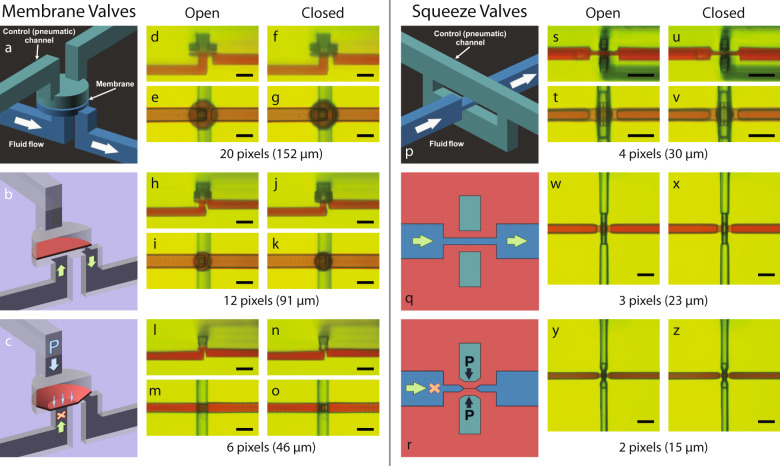
3D printed membrane valves and squeeze valves. **a** Schematic diagram of membrane valve geometry with cut-away schematics showing a membrane valve in (**b**) open and (**c**) closed states depending on pneumatic pressure applied through the control channel. **d**–**o** Side- and top-view microscope images of (**d**–**g**) 20 pixel, (**h**–**k**) 12 pixel, and (**l**–**o**) 6 pixel diameter valves in their open and closed states. **p** Schematic diagram of squeeze valve geometry with cross-section diagrams (rotated 90^∘^) showing a squeeze valve in (**q**) open and (**r**) closed states depending on pneumatic pressure applied through the control channel, which squeezes together to close the flow channel. **s**–**z** Microscope images of (**s**–**v**) 4 × 4 pixel (side- and top-view), (**w**,**x**) 3 × 3 pixel (top-view), and (**y**,**z**) 2 × 2 pixel (top-view) valves in their open and closed states. All scale bars are 100 μm.

A membrane valve, as shown in (Fig. 1a–c), is composed of a control (pneumatic) chamber and fluid chamber separated by a thin membrane^7,20,22^. When the control chamber is pressurized, the membrane deflects until it covers the channel connected to the center of the bottom of the fluid chamber, blocking fluid flow and therefore closing the valve (Fig. 1c). When the pneumatic pressure is released, the stretched membrane returns to its original position (Fig. 1b), opening the valve. Two channels connect to the control chamber, one of which goes to an external pressure source, while the other facilitates flushing unpolymerized resin left inside after the printing process. Blocking this second channel before device use^20^ insures only one pneumatic connection to a given control chamber during operation.

A critical limiting factor when miniaturizing 3D printed valves is the 3D printer x–y resolution. Our 3D printer has a 7.6 μm pixel pitch such that this defines all microfluidic features by projected images comprised of 7.6 μm square pixels^16^. One consequence is that as the diameter of the cylindrical valve region is reduced to relatively few pixels, the edges of the nominally circular membrane become more pixelated.

Figure 1d–o shows side and bottom views of 3D printed membrane valves with diameters ranging from 20 pixels (152 μm) down to 6 pixels (46 μm) under open and closed conditions. In all cases, the valves function as designed to block or allow fluid flow depending on whether the control chamber is pressurized or not. Careful comparison of the cross-section micrographs, (d,f), (h,j), and (l,n), show clear deflection of each membrane when pressurizing the control chamber to close the valve. In particular, note in Fig. 1n the remarkable amount of deflection exhibited by the thin 6-pixel diameter valve membrane compared to Fig. 1(l).

**Fig. 2:**
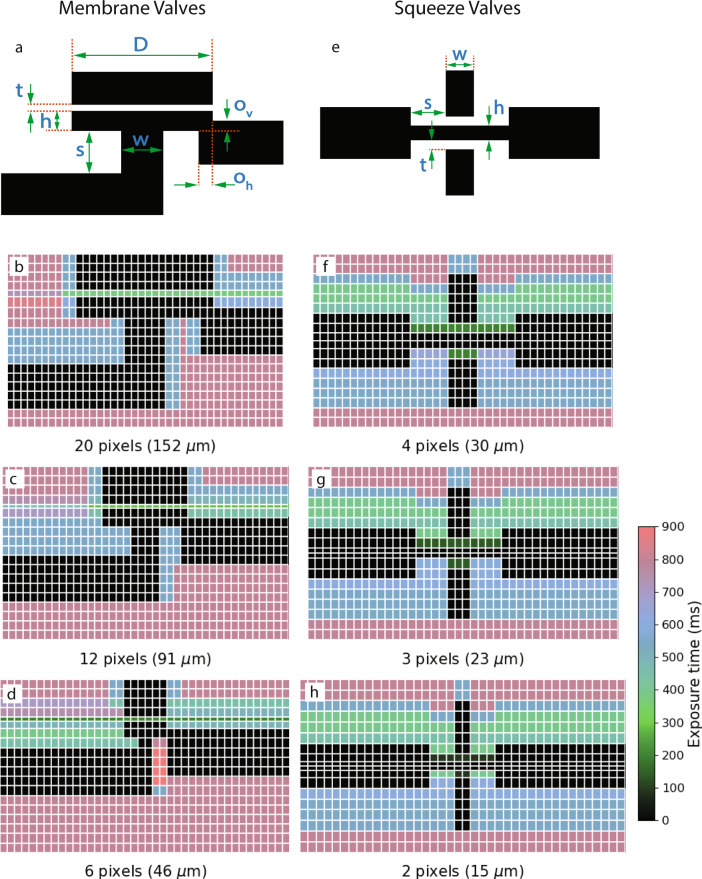
Deliberate exposure time and layer thickness variation in 3D printing process. **a** Membrane valve designed dimension parameters, (**b**–**d**) membrane valve exposure times. **e** Squeeze valve designed dimension parameters, (**f**–**h**) squeeze valve exposure times. Tables 1 and 2 specify the variable layer thicknesses used for each type of valve.

Fabrication of all of the valves shown in Fig. 1 used our versatile 3D printing approach which is enabled by the complete control we have over all aspects of our custom 3D printer hardware and software, and which allows us to create polymerized features optimized for their designed function at the scale of relatively few pixels and layers. For example, Fig. 2a and Table 1 specify the design dimension parameters used for creating the different size membrane valves. Note in particular the different layer thicknesses and exposure times for the membrane layer, which in the case of the 6-pixel diameter valves is designed to be 4 μm thick.

**Table 1:** Membrane valve parameters and closing pressures.

Diameter (pixels)	Diameter (D)	Membrane layer thickness (t)	Fluid chamber height (h)	Center channel width (w)	Center channel height (s)	Edge channel vertical overlap (*o*_*v*_)	Edge channel horizontal overlap (*o*_*h*_)	Exposure Time (ms)	Closing Pressure (psi)
20	152	7	24	46	50	12	15	380	25
12	91	4	20	30	30	10	8	280	14
6	46	4	18	15	10	10	0	200	9

All dimensions in microns unless otherwise noted. Symbols refer to Fig. 2(a).

The pressure required to close a membrane valve depends on the membrane’s area (*πD*^2^/4), the fluid chamber height (h), the cross-section area (*w*^2^) of the channel to be blocked, and the membrane thickness. The latter is contingent on the optical dose the membrane receives, since there is unpolymerized resin in the region behind the membrane that polymerizes as the dose increases^15,16^. The dose is proportional to the time the membrane is exposed by the 3D printer light source. For the fabrication parameters given, Table 1 shows the measured minimum pressure required to fully close the valves: as small as 9 psi for the 6 pixel valve.

Figure 2b–d show cross-sectional views of each of the membrane valves. The vertical grid lines delineate the 7.6 μm pixels, while the horizontal grid lines indicate the build layers, typically 10 μm in thickness, except for the membrane and fluid chamber layers. As seen in the figures, the optical dose that each of the non-void pixels receives is not uniform within a layer. This exposure difference mainly has to do with the fact that the void features can end up polymerized if the regions on top of or next to them are overexposed^15,16^. The different doses within a layer are achieved by exposing multiple overlapping images, each with their own exposure time, to a single layer to control the dose on a pixel-by-pixel basis. This approach permits us to not overexpose on top of or next to void features while maintaining bulk chip strength by applying a higher optical dose to bulk regions.

Numerous trade-offs determine specific choices for exposure times and layer thicknesses in the broad design parameter space available for a given valve size. For example, we can reduce the actuation pressure for 20 pixel valves to below 15 psi if we decrease the membrane thickness and fluid chamber height. However, this can make the membranes too weak for devices where we have many closely spaced void features (due to the printing process) and lower their response time, which we require to be fast (≤15 ms) if we want to use them as part of pumps, which we show in the next section.

The membrane-based pumps and mixers used for serial dilution in Sect. *Integrated 10-stage 2-fold serial dilution devices* are based on 20 pixel valves, since the investigation of 12 and 6 pixel valves occurred after testing the serial diluter. We have included videos of membrane valve operation as Supplementary Videos 1 and 2 (video descriptions are in Supplementary Note 1). We also show in Supplementary Note 2 and Supplementary Fig. 1a vertically oriented 3D printed membrane valve, where its membrane comprises 30 stacked layers to create a 300 μm diameter membrane, which is equivalent to the size of the horizontal 40-pixel diameter valves we reported in Ref. ^20^.

The geometry of our squeeze valve is shown in (Fig. 1p–r). As illustrated in (Fig. 1p), the significantly reduced height of the fluid channel passes through a vertically split control channel. The top and bottom of the fluid channel are separated from each control channel by a thin layer of polymerized resin. Table 2 shows the resin layer thickness and exposure time used to fabricate these small membranes, highlighting our approach to 3D printing. As shown in the top view micrographs (Fig. 1t–z), the membranes have a square geometry that varies from 4 × 4 pixels (30 μm × 30 μm) to 2 × 2 pixels (15 μm × 15 μm). The widths of the overlapping fluid and control channels define the valve membranes, which are oriented at 90^∘^ relative to each other. This arrangement makes the squeezed region a cuboid with the top and bottom defined by square membranes.

**Table 2:** Squeeze valve parameters.

Width (pixels)	Width (w)	Separation (s)	Membrane layer thickness (t)	Fluid channel height (h)	Exposure time (ms)	Closing pressure (psi)
4	30	38	10	16	200	38
3	23	30	10	10	140	26
2	15	23	7	8	90	12

All dimensions in microns unless otherwise noted. Symbols refer to Fig. 2(e).

When the control channel is pneumatically pressurized, the thin top and bottom membranes deflect, as illustrated in Fig. 1r, closing the valve. When the control channel pressure is released, the membranes snap back, as illustrated in Fig. 1q, opening the valve. In (Fig. 1t–z), the valve area appears red when a valve is open because of the aqueous red dye solution in the fluid channel. There is no red color in the valve region when a valve is closed because the red dye solution has been squeezed out into the adjacent fluid channel. Supplementary Video 3 shows a squeeze valve in operation.

As shown in Table 2, 2-pixel valves take as little as 12 psi to close. We could also successfully 3D print and test 4-pixel squeeze valves with a single control channel (the top channel in Fig. 1p). However, the pressure required to close these valves was over 60 psi. Hence, splitting the control channel to pass both above and below the fluid channel reduces the required actuation pressure by creating top and bottom membranes that undergo less deflection to close the channel compared to a single top membrane.

Effectively fabricating such 3D geometries is enabled by our ability to independently specify layer thicknesses and pixel doses. Figure 2e–h shows a cross-sectional view of each of the squeeze valves and the exposure time applied to each pixel. Table 2 reports relevant geometric parameters. Similar to the membrane valves in Fig. 2, we make extensive use of the dose and layer thickness control capability inherent in our 3D printing approach to finely control the polymerization of the 3D printing process to achieve the desired device structures.

### Characterization of 3D printed pumps

When opening an initially closed valve, a specific volume of fluid is drawn from the attached fluid channels into the enlarged fluid chamber volume created by movement of the valve membrane. The opposite happens when closing an initially open valve. We can use this fluid displacement phenomenon to create a pump by connecting an additional valve to the fluid input channel and another to the fluid output channel, and appropriately synchronizing the opening and closing of these valves in conjunction with the center valve to control which fluid channel the central valve draws fluid from and to which it expels fluid.

**Fig. 3:**
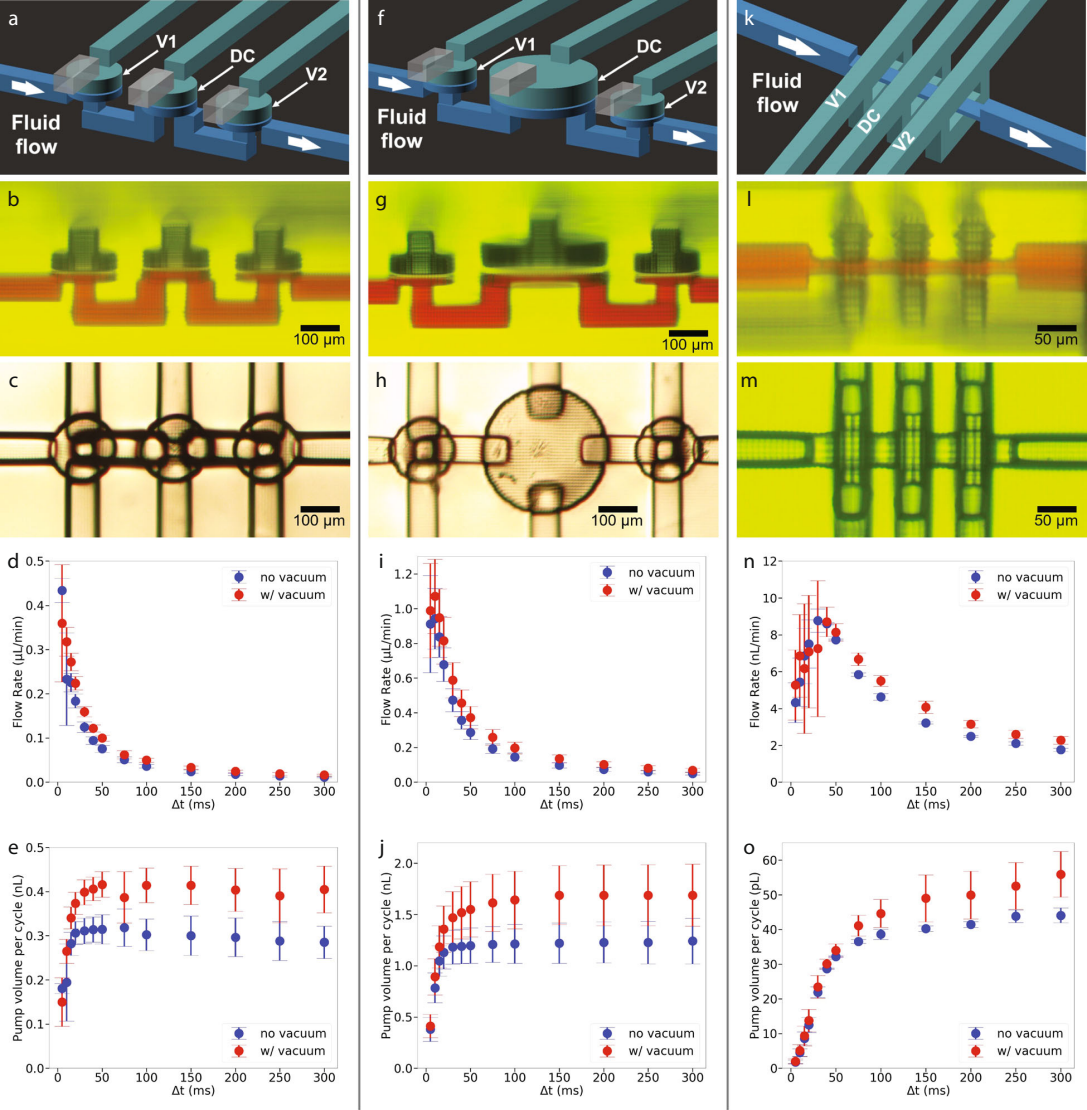
3D printed membrane and squeeze valve pumps. **a**, **f**, **k** Schematic diagrams. **b**, **g**, **l** Side view microscope photos. **c**, **h**, **m** Bottom view microscope photos. **d**, **i**, **n** Volumetric flow rate as a function of the pump phase interval, Δt. **e**, **j**, **o** Pump volume per cycle as a function of pump phase interval. All graphs were obtained from testing at least three different pumps.

Two 20-pixel diameter membrane valves and a 20-pixel DC (defined in Sect. *Pump measurements*) form the first pump in Fig. 3(a–e). Table 1 shows the valve parameters in the first row. The DC had identical parameters except its fluid chamber height is 31 μm instead of 24 μm to increase the amount of fluid expelled during each pump cycle. Note that the increased fluid chamber height compared to the valves is visible in the side view micrograph in Fig. 3(b) (i.e., the height of the red fluid chamber is larger for the DC). The measured pump flow rate, shown in Fig. 3d, is a function of the phase time, Δt, defined in Sect. *Pump measurements*. For example, a 50 ms phase time (250 ms pump cycle) results in a volumetric flow rate of close to 0.1 μL/min. Supplementary Video 4 shows a pump in operation.

The second pump (Fig. 3f–j) is the same as the first example, except the diameter of the DC increases from 20 pixels to 40 pixels and the membrane consists of two 6 μm layers while the fluid chamber height remains at 31 μm. The purpose of the increased DC diameter is to increase the pump flow rate by increasing the fluid volume expelled during each pump cycle^13^. We expected the volume to increase fourfold since the DC diameter increased twofold and the fluid chamber height remained the same. These data show that for a 50 ms phase time no vacuum case, the pump volume per cycle increased as expected from 0.3 nL to 1.2 nL, and the flow rate increased from 0.075 μL/min to 0.3 μL/min. Supplementary Video 5 illustrates the increased flow rate due to the larger DC compared to Supplementary Video 4.

The third pump (Fig. 3k–o) consists of three 4-pixel squeeze valves. Note the compact size of the pump: 182 μm ×167 μm × 136 μm = 0.0041 mm^3^, which is achieved in part by separating the valves by only 6 pixels (46 μm) along the flow channel. Commensurate with its small size, its generated flow rate is also low. For example, Figs. 3n, o show that for a phase time of 50 ms, the volumetric flow rate is ~8 nL/min, while the fluid volume expelled during each pump cycle is 30 pL. To get a sense of scale for the generated volumetric flow rate, we can compare this to the sweat generation rate for a human, which is 1 nL/min per sweat gland, so the pump flow rate is equivalent to what is produced by eight sweat glands^23^. Operation of a squeeze valve pump is demonstrated in Supplementary Video 6.

### Diffusion mixing

As is well-known, fluid mixing is a challenge at the low Reynolds numbers typical of microfluidic device operation since fluid flow is laminar^24,25^. Over several decades, various passive and active mixing strategies have been demonstrated. Comprehensive reviews of such strategies can be found in Refs. ^24–26^. In this paper, we take a different approach to mixing that relies solely on one-dimensional (1D) diffusion in a narrow channel that is specifically designed for fast mixing times (~1 s) in a short length (<1 mm).

**Fig. 4:**
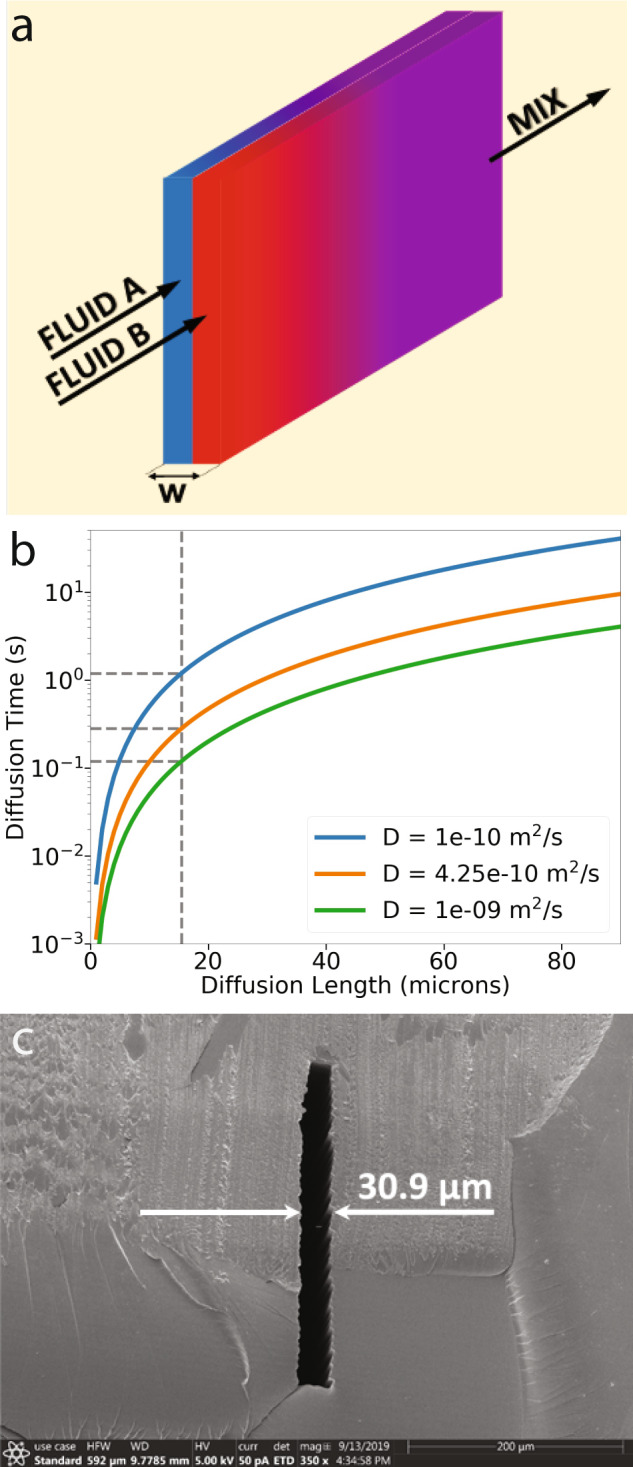
Fast diffusion mixing. **a** Two thin fluid sheets in a narrow vertical channel. **b** Diffusion time for example molecules over a 15 μm diffusion length in an aqueous solution. *D* is the diffusion coefficient. Blue = 30 kDa protein, Orange = fluorescein, Green = dissolved gas molecules. **c** Cross-section scanning electron microscope (SEM) image of narrow 3D printed diffusion mixing channel.

The time required to diffuse, *t*_*D*_, over a distance, *l*, is given by^27^

1 $${t}_{D}=\frac{{l}^{2}}{2D}$$

where *D* is the diffusion coefficient of the molecular species in solution. Clearly, the shorter the diffusion distance, *l*, the shorter the time it takes to mix. For our mixer, we therefore choose to use a tall, high aspect ratio channel, as shown schematically in Fig. 4a, in which we load two fluids side-by-side such that the distance, *l* = *w*/2, over which molecules from one fluid need to diffuse into the second fluid is small. If the dwell time of the fluids in the channel is greater than *t*_*D*_, diffusion-based mixing will occur. For example, Fig. 4b shows the diffusion time as a function of diffusion length for several representative diffusion coefficients. In the fluorescein case (orange curve), the needed dwell time is several hundred milliseconds if the diffusion length is 15 μm. Figure 4c shows an SEM image of the cut cross-section of a 4-pixel wide high aspect ratio channel we designed for mixing given our 3D printer capabilities. The average measured width, *w*, is 30.9 μm, which results in a diffusion length of just over 15 μm, corresponding to diffusion times ranging from 0.12 to 1.2 s for the diffusion coefficients shown in Fig. 4b.

**Fig. 5:**
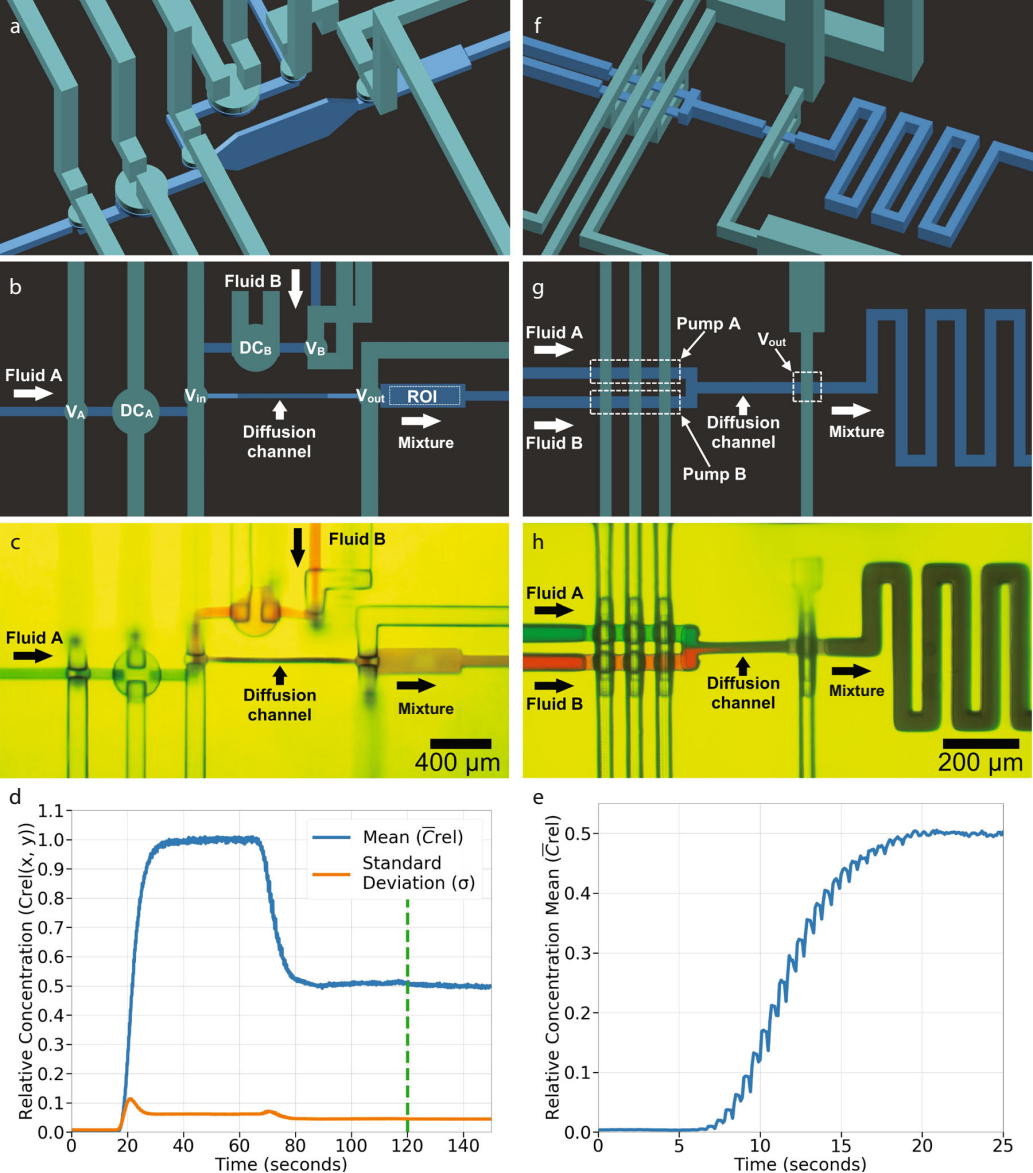
Single stage 1:1 mixer. **a**–**e** Membrane valve-based pump version: schematic diagram (**a**) perspective view and (**b**) top view, (**c**) microscope photo, (**d**) mixing test, and (**e**) time to equilibrium test. Note in (**d**) that the mean, ${\bar{C}}_{rel}$, and standard deviation, *σ*, of the relative concentration are both plotted on the left vertical axis. **f**–**h** Squeeze valve-based pump version: schematic diagram (**f**) perspective view and (**g**) top view, (**h**) microscope photo.

We are particularly interested in mixing two fluids in equal amounts to create a 1:1 mixture. We can create a simple but effective 1:1 diffusion-driven mixer by connecting the outputs of two identical pneumatically actuated pumps to the high aspect ratio diffusion channel such that the fluids form adjacent sheets as schematically illustrated in Fig. 4a. Figure 5a–c shows a 1:1 mixer based on two membrane valve-based pumps with 40-pixel DCs like in Fig. 3f. Both pumps have the same outlet valve in common^22^, which is the inlet valve, *V*_*in*_, to the diffusion mixing channel. An extra valve, *V*_*out*_, is added after the diffusion channel to ensure that the already mixed fluid that has exited the diffusion channel does not interact with the fluids inside the channel while they are still mixing. The diffusion channel is 950 μm long and 370 μm tall with tapered entrance and exit heights. The total diffusion channel volume is 8.95 nL. Section *Mixer measurements* describes the timing sequence and mixing time tradeoffs.

Figure 5c shows a mixer snapshot while it mixes red and blue dyes to illustrate mixing efficacy visually. Figure 5d shows a typical quantitative measurement, which consists of first pumping only a non-fluorescent fluid (Fluid B) to obtain the minimum fluorescence intensity across the ROI (0–15 s in Fig. 5d), followed by pumping only a fluorescein solution (Fluid A) to obtain the maximum fluorescence intensity (40–65 s). Since we have separate control over each input pump, this enables these types of control experiments. Next, we pump both fluids using the nine-phase cycle in Sect. *Mixer measurements* to measure the fluorescence of the mixed fluids (90–120 s). At 120 s, we stop the pumps and close all valves. This approach allows us to monitor the standard deviation of the mixed fluorescein concentration in the ROI as a function of time (120–140 s). The evolution of the standard deviation over time is a measure of how well mixed the fluid is, with a well-mixed fluid showing no change over time and a poorly mixed fluid showing a decrease over time^13^. Note that the standard deviation (orange curve in Fig. 5d) shows no change over time, indicating that the fluorescein 1:1 dilution is well-mixed.

Finally, the time evolution of the mixed fluorescein concentration in the ROI is shown in Fig. 5(e) for a starting state with no fluorescein in the 1:1 mixer module. As can be seen, it takes ~20 s for the fluorescein concentration in the output channel to reach a steady state, with the majority of this time needed for the pumps to displace the dead volume in the fluorescein pump, mixer channel, and outlet channel. We note that the fluid in the mixer channel is static during 8 out of the 9 phases used for each period of the mixer cycle (Table 3) such that static diffusion is the dominant mixing mechanism. Also note that on the way toward steady state, there is a periodic variation in the concentration. This variation is due to fluid downstream of the ROI, which has lower fluorescein concentration before reaching steady state, being drawn back into the ROI when valve V_*in*_ opens at t_6_ in Table 3. This fluid is expelled from the ROI when DC_*A*_ and DC_*B*_ are actuated at t_7_ and replaced with a higher concentration fluorescein mixture as the 1:1 mixer moves toward a steady state concentration output.

**Table 3:** Timing sequence for membrane valve-based mixer.

	t_0_	t_1_	t_2_	t_3_	t_4_	t_5_	t_6_	t_7_	t_8_
V_*A*_	●		●	●	●	●	●	●	●
DC_*A*_	●							●	●
V_*B*_	●	●	●		●	●	●	●	●
DC_*B*_	●	●	●					●	●
V_*in*_	●	●	●	●	●	●			●
V_*out*_	●	●	●	●	●				

Black circle: valve closed, No circle: valve open.

Figure 5f–h shows an alternate, much smaller (compare scale bars in Fig. 5c, h) 1:1 mixer module that uses pumps based on squeeze valves. The main difference is that for this design, we arranged the pumps such that they share the same control lines. This approach reduces the number of phases required for a complete actuation cycle and means the two fluids cannot be pumped separately. The seven-phase valve timing logic sequence that we use to operate the mixer is shown in Supplementary Table 2. The number of phases is reduced by two compared to the 1:1 mixer based on membrane valves. We found that the single-stage mixers with squeeze valves performed comparably to the membrane-valve based mixers.

### Integrated 10-stage twofold serial dilution devices

**Fig. 6:**
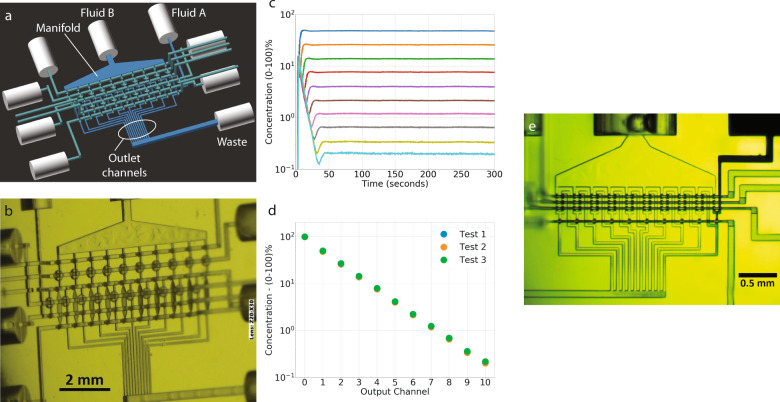
10-stage twofold serial diluters. **a** CAD drawing and (**b**) microscope image of membrane valve-based serial diluter. **c** Normalized fluorescein concentration as a function of time for all ten output channels (outputs 1–10) and the input concentration (output 0). **d** Steady state normalized fluorescein concentration at each output channel for three repeated tests. **e** Microscope image of 10-stage twofold serial diluter made with squeeze valve-based 1:1 mixer modules.

Using ten compact 1:1 mixer modules arranged in series, we designed a 10-stage serial dilution module that provides simultaneous twofold dilution of a starting sample to ten outputs with concentrations covering three orders of magnitude. Each 1:1 mixer module comprises a single twofold dilution stage in which its output is equally split between an output channel of the serial diluter and the input to the next twofold dilution stage. Figure 6a shows a 3D CAD drawing of one of our designs based on membrane valves. The fluid to be serially diluted is introduced at the Fluid A input, and the diluent is presented at the Fluid B input. Note that the Fluid B input is attached to a large manifold. The diluent inputs for each twofold dilution stage draw fluid from this manifold. The figure also shows the ten outlet channels from the twofold dilution stages, all connected to a waste outlet since this design is intended only for proof-of-principle and measurement of the ten simultaneously generated output concentrations. The other cylindrical tubing connections in the CAD design are pneumatic control inputs for the various valves and DCs. The serial dilution module contains a total of 40 valves and 20 DCs. These are organized into 20 pumps, two for each twofold dilution stage, and 10 diffusion channel outlet valves.

Figure 6b shows a microscope image of a fabricated device. The camera is at an angle to the chip’s surface normal so that the narrow diffusion channels can be seen more clearly. As illustrated in Supplementary Fig. 3b, we typically fabricate two 10-stage serial diluters per chip for purposes of testing. Note that a single 10-stage serial dilutor with its chip-to-world interconnects uses ~1/3 of the chip real estate, leaving the rest of the chip available to place fluidic components to use the ten serial diluter outputs in parallel to ultimately permit conducting a complete dose-response assay on a single chip.

Figure 6c shows the normalized fluorescein concentration for each of the ten serial diluter output channels as a function of time during a 10-stage serial diluter startup. Details are discussed in Sect. *Serial dilutor measurements*. The important observation gained from Fig. 6c is that the fluorescein concentrations reach a steady state in all the output channels in <50 s of operation, dictated by the time needed for the entire dead volume of the dilutor to be replaced by the pumps. Figure 6d shows the steady state normalized fluorescein concentration in each output channel where output 0 is the undiluted fluorescein solution. As expected, the output concentrations are linear on a log scale and cover three orders of magnitude of concentration. A video of the 10-stage diluter conducting a serial dilution of black dye with water is shown in Supplementary Video 7 and described in Supplementary Note 5. Consistent with Fig. 6c, the output channels in the video are seen to reach a steady state within 50 s.

We also created a 10-stage serial diluter as a proof-of-principle using the 1:1 mixer squeeze valve module shown in Fig. 5h. Our motivation was to demonstrate the miniaturization potential of components made with squeeze valves. A fabricated 10-stage serial diluter is shown in Fig. 6e. Note the degree of miniaturization where the x–y footprint of the serial diluter is only 2.2 mm × 1.1 mm. A video of the serial diluter in operation is included as Supplementary Video 8 and described in Supplementary Note 5. While the squeeze valve-based version of the serial diluter performed comparably to the larger membrane valve-based version, it took ~2.5 min to reach steady state compared to the membrane-valve serial diluter because of the smaller amount of fluid displaced by the squeeze valves compared to the volume of the output channels. We therefore opted to use a membrane valve version for the serial diluter in our dose-response assay in the next section because of the shorter time to achieve a steady state. Nonetheless, the preliminary proof-of-principle device shown in Fig. 6e indicates the potential of our approach to 3D printing for microfluidic device miniaturization and integration.

### Digitonin assay

**Fig. 7:**
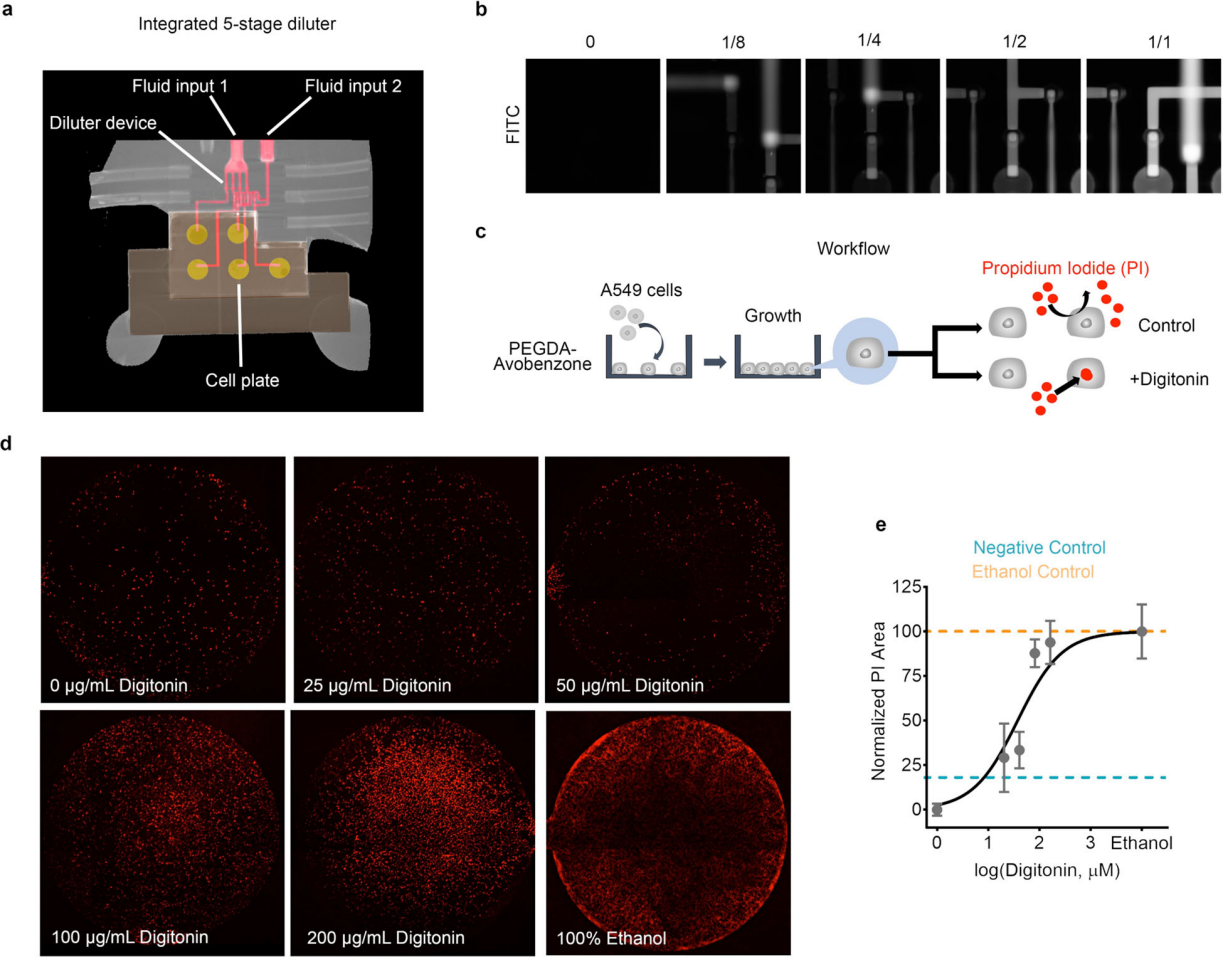
Digitonin permeabilization assay. **a** μCT image of 3D printed 5-stage diluter integrated with 3D printed cell plate. **b** Fluorescence images of serially diluted fluorescein. The experiment was repeated three times and all show similar results. **c** Cell treatment workflow. **d** Whole well images of differentially treated A549 cells with propidium iodide as a marker (red). The treatment fluid was 100 μg/mL digitonin and 2 μM propidium iodide in DMEM/F12 while the control fluid was 2 μM propidium iodide in DMEM/F12. 100% ethanol was used as a positive control for the whole experiment. **e** The semi-log dose response-curve was derived from the experiment. Responses were quantified through the measurement of propidium iodide area relative to the total cell area (DIC). Bounds were set at 0 μg/mL digitonin (0 response) and 100% ethanol (100% response). Values were derived from *n* = 3 independent experiments. Error bars denote standard deviation.

As a proof-of-principle illustration of integrating a multi-stage serial diluter with a dose-response assay, we designed a test system comprised of two 3D printed chips as illustrated in Fig. 7a and Supplementary Fig. 5. One chip (Supplementary Fig. 5b) simultaneously generates five outputs with relative concentrations of 1/1, 1/2, 1/4, 1/8, and 0 from two input fluids, which we term the treatment fluid (Fluid input 1) and the diluent fluid (Fluid input 2). A concentration of 0 represents an output with only the diluent and serves as a negative control. The second chip is a cell plate containing five microwells that are open on its upper surface and small waste channels. Each microwell is 1.78 mm in diameter (Supplementary Fig. 5a), holds 1 μL, and is surrounded by a microgasket^20^. The two chips are clamped together with a custom holder to form a leak-tight seal between the serial diluter chip and the cell plate chip. Each of the five fluid outputs of the serial diluter chip is connected to a single cell plate microwell as indicated in Supplementary Fig. 5c. The serial diluter chip is fabricated with our NPS-PEGDA resin^16^, while the cell plate chip is fabricated with our biocompatible resin reported in Ref. ^28^.

Micro-computer tomography (μCT) was used (Supplementary Fig. 5) to image the plates. An iodine-potassium iodide (I2/KI) solution was perfused into the fluidic channels, creating high contrast compared to the bulk 3D printed polymer. For example, the pink channels in Fig. 7a illustrate how the diluter outputs are routed to the microwells (yellow circles) on the cell plate chip. In addition, fluorescein was used to confirm the diluter mechanism by presenting a fluorescein solution at the treatment input and cell culture media DMEM/F12 at the diluent input. Figure 7b shows the expected diminishing fluorescence in the output channels for each serial dilution stage and the lack of fluorescence for the negative control stage.

As a biological proof-of-concept using this two-chip dose-response assay system, we show selective permeabilization using digitonin, a steroidal saponin. The use of digitonin in selective permeabilization has been shown in chromaffin cells^29^, astrocytes^30–32^, and cancer cells such as A549^33,34^ among others. The cell treatment workflow is shown in Fig. 7c in which lung adenocarcinoma A549 cancer cells were seeded into the microwells of a cell plate chip that had been surface modified with a 6 min oxygen plasma treatment^28^. The cells were then grown for 3 days until confluence, after which the cell plate chip was integrated with a serial diluter chip. A cell impermeant nuclear stain, propidium iodide, was used as a marker of permeabilization. As shown in Fig. 7d and Supplementary Fig. 6, we observed increasing nuclear stain with increasing digitonin concentration. Data were collected and plotted on a semi-log dose-response curve using 0 μM digitonin as the baseline and 100% ethanol as the maximal response (Fig. 7e). We estimate *EC*_50_ as 37.5 μM (24.0–57.2 μM, CI = 95%, *R*^2^ = 0.83). Previously, various researchers reported a range of digitonin concentrations used in cell membrane permeabilization experiments. Experiments on bovine chromaffin cells reported the use of 30 μM digitonin to achieve 90% cell permeabilization^35^. Moreover, in astrocytes^30–32^, 20–30 μM digitonin was used while 20–40 μM digitonin was used in A549 cells^33,34^ to achieve at least 75% plasma membrane permeabilization. Though, to our reading, definitive EC50 derivation for digitonin-based permeabilization in A549 has not yet been established. Alternatively, an EC50 value of 65.79 μM for digitonin has been shown in CHO cells using an Aequorin reporter assay^36^.

## Discussion

Results reported in this paper show that our generalized 3D printing approach is effective for fabricating tiny active components such as valves and pumps on a size scale that until now has only been available using conventional lithographic methods. Moreover, we have shown that our approach is also effective in integrating such small active components into more sophisticated 3D functional structures such as active mixers and serial dilutors. This approach opens the door to replacing expensive and time-consuming cleanroom processes and equipment with fast and much less expensive 3D printing, which would revolutionize microfluidic device development^10^. Moreover, in principle any DLP-SL 3D printer manufacturer could implement the generalized 3D printing approach discussed in this paper as long as the raw projected image resolution is sufficient (7.6 μm in our case). To facilitate the broad adoption of our generalized 3D printing approach and the ability to share device designs, we have open-sourced a 3D print file specification based on the standard JSON (javascript object notation) format that incorporates all of the features of our 3D printing approach, which we have made freely available on github.com (10.5281/zenodo.5199514, https://github.com/3D-Printing-for-Microfluidics/3D_printer_json_print_file)^37^.

A common concern regarding high resolution DLP-SL for microfluidics is the trade-off between resolution and image area: the higher the resolution (i.e., the smaller the projected pixel size), the more limited the image area and therefore the smaller the maximum device x–y footprint. This perceived shortcoming can be marginally compensated using a larger format micromirror array, such as a 4 megapixel (MP) array instead of a 2 MP array. We use a 4 MP micromirror array in our custom 3D printers, resulting in an image area of 19.5 mm × 12.2 mm, which is twice as large as for a 2 MP format, but may still be too small for some applications. A straightforward method to overcome this trade-off is to stitch images into a larger area multi-image mosaic for each layer^38^. With this approach, microfluidic devices the size of a well plate could be possible. However, for many applications, this may be unnecessary. We argue that it may be more attractive to make 3D printed microfluidic devices as small as possible so that many devices can be fabricated in a single 3D print run to take advantage of parallel fabrication for manufacturing. For example, consider Supplementary Fig. 7 in which we printed 117 identical individual chips in a single 3D print run as described in Supplementary Note 7. Each chip contains a pump with compact chip-to-chip interconnects^20^ to facilitate integration with a more extensive reusable 3D printed chip with the required bulky chip-to-world interconnects and additional microfluidic functionality. Focusing on parallel printing of such small chips but with sophisticated functionality offers a path to manufacturability and componentized assembly of more complex devices. Examples include well plate-sized constructs in which a number of highly compact 3D printed devices are integrated onto a larger, lower resolution piece that combines with a well plate, or devices that integrate directly into the well plate itself. In summary, our generalized 3D printing approach opens many new possibilities for microfluidics beyond those available with traditional lithography-based fabrication methods. We hope that our approach will be broadly adopted to rapidly advance microfluidics research and applications.

## Methods

### Materials

For 3D printing we use a custom photopolymerizable resin which consists of poly(ethylene glycol) diacrylate (PEGDA, MW258) with a 1% (w/w) phenylbis(2,4,6-trimethylbenzoyl)phosphine oxide (Irgacure 819) photoinitiator and a 2% (w/w) 2-nitrophenyl phenyl sulfide (NPS) UV absorber, details of which are provided in Refs. ^13,16,20^.

Uranine powder (40%) was procured from Fischer Science, and Macron Fine Chemicals supplied sodium hydroxide pellets.

### 3D printing and sample preparation

The custom 3D printer used in this paper has a 385 nm LED light source and a pixel pitch of 7.6 μm in the projected image plane. We refer to it as the High Resolution 2 3D printer (also referred to as the Generation 2 3D printer in^39^). It is the next generation of custom 3D printer originally reported in Ref. ^16^.

For 3D printing substrates, we use 25 mm square silanized glass slides. Slides are first rinsed with acetone and isopropyl alcohol (IPA) and then immersed in toluene mixed with 10% 3-(trimethoxysilyl)propyl methacrylate for 2 h. After silanization, we store the glass slides in fresh toluene inside a closed container until use, ranging from under an hour to several weeks. Unless otherwise noted, all 3D prints reported in this paper are fabricated with a layer thickness of 10 μm and an exposure time of 900 ms. The image plane irradiance is 21.2 mW⋅cm^−2^ with an LED source spectrum as reported in Ref. ^16^.

### Pump measurements

Pumps are operated with the five-phase valve opening and closing sequence shown in Supplementary Table 1^22^. A graphic illustration of the pump sequence is shown in Supplementary Fig. 2 for a pump comprised of three 4-pixel squeeze valves to pump fluid from an inlet valve (V1) to an outlet valve (V2). The phase time, Δ*t* = *t*_*i*+1_ − *t*_*i*_, is defined as the time for a single phase in the five-phase pump sequence in Supplementary Table 1.

Since at any single phase of the pumping cycle either V1 or V2 is closed, the center valve does not have to block fluid flow when it is actuated. For pumps based on membrane valves, we therefore position both fluid channels at the edges of the center valve’s fluid chamber so that the structure can only displace fluid and not block fluid flow^22^. We call this modified structure a displacement chamber (DC). It defines the fluid volume pumped in each pump cycle^13^.

To measure pump volumetric flow rate as a function of phase time, we recorded video at 240 frames per second using a cell phone camera attached to a microscope while pumping a dye solution through an initially empty microfluidic channel with known dimensions. This approach allowed us to track the fluid meniscus frame-by-frame with a custom Python script to determine the volumetric flow rate^13^. The inlet fluid and the pump outlet were kept at the same height as the pump to permit the determination of fluid flow for zero back pressure.

Data are shown in Fig. 3d for two cases relative to how a DC is opened, i.e., how the membrane is transitioned from its deflected closed state to an open state. The “no vacuum” case refers to the control chamber being switched from positive pressure to atmospheric pressure such that the main restoring force on the membrane is its mechanical relaxation from a stretched to an unstretched condition. The “with vacuum” case consists of switching from a positive pressure in the control channel to negative pressure, in which case there is an additional pneumatic restoring force for the membrane^22^. As shown in Fig. 3e, application of vacuum results in an approximately 30% higher volume expelled during each pump cycle, which is due to the negative pressure in the control chamber causing the membrane to deflect up into the control chamber, thereby increasing the volume of fluid pulled into the fluid chamber. For example, for a 50 ms phase time, the fluid volume increases from ~0.3 to 0.4 nL.

### Mixer measurements

Table 3 shows the nine-phase timing sequence we use to operate a 1:1 mixer based on membrane valves. Referring to Fig. 5b, Fluid A is first pulled into DC_*A*_, followed by Fluid B into DC_*B*_, after which V_*in*_ and V_*out*_ are opened, and fluid from both DCs is simultaneously pushed into the diffusion channel, then the process repeats. For a 50 ms phase time, a single nine-phase mixer period is 450 ms. We deliberately designed the volume of the diffusion channel to be nearly four times the volume pumped into it by both pumps during a single nine-phase period such that the average fluid dwell time in the channel is nearly four nine-phase mixer periods (i.e., 1.7 s). Note that this permits mixing even for proteins that are 10’s of kDa. Thus, mixing larger molecules with smaller diffusion coefficients can be performed in the same mixer structure by increasing the dwell time in the diffusion channel. This adjustment can be accomplished by decreasing the flow rate into the diffusion channel by increasing the phase time. Alternatively, the diffusion channel dwell time can be increased by redesigning the 1:1 mixer module with a larger volume diffusion channel having an increased height or length (or both) and/or by decreasing the size of the pump DCs to reduce the effective flow rate into the diffusion channel for a given phase time.

To quantitatively measure mixer performance, we used fluorescence measurements with a dilute fluorescein solution. We focused a microscope on the output channel region of interest (ROI) just to the right of the diffusion channel in Fig. 5b and acquired fluorescence images of the fluorescent fluid, such that the obtained image intensity values were proportional to the concentration of fluorescein inside the ROI. We used a video analysis method to measure the effectiveness of diffusion channel mixing based on an analysis of the fluorescence standard deviation^13^. We used 100 μM fluorescein in 0.1M NaOH and 0.1M NaOH as fluid inputs A and B. A phase interval of 50 ms (450 ms per complete mixer cycle) was sufficient to mix the fluorescein solution thoroughly. In addition, pressures of 25 PSI and 10 PSI were used to actuate valves and DCs, respectively. An Olympus IX73 fluorescence microscope and ORCA-Flash 4.0 camera (Hamamatsu) were used for fluorescein fluorescence measurements.

### Serial dilutor measurements

Serial diluter characterization was also done with 100 μM fluorescein in 0.1M NaOH diluent and 0.1M NaOH as fluid inputs A and B, respectively. Fluorescence in the serial diluter output channels is normalized to that of the undiluted sample fluid and converted to percent.

For the experiment shown in Fig. 6c, the initial state of the serial diluter (*t* = 0) is diluent fluid in all the diluent pumps for all stages and outlet channels, and undiluted fluorescein solution (denoted subsequently as 100% concentration) in all the fluorescein solution pumps in each stage. This situation occurred because the experimental sequence was (1) pump 100% fluorescein solution through the fluorescein pumps and into the output channels to get a maximum fluorescence baseline, followed by (2) pump 100% diluent through the diluent pumps and into the outlet channels to get a minimum fluorescence baseline, and then (3) normal operation of the serial diluter in which all the pumps are used. The net result is that right after starting step (3) at *t* = 0, there is a spike in fluorescence as the 100% fluorescein solution is cleared from each stage’s fluorescein pump, followed by a decrease in fluorescence, and then a rise until steady state is reached.

Microscopy at non-normal incidence was performed with a Keyence VHX-970 Digital Microscope.

### Dose-response assay measurements

#### O_2_ plasma treatment

Cell plates were exposed to O_2_ plasma for 6 min using a parallel-plate plasma etcher (Technics PlanarEtch II) at 200 W with 10 sccm O_2_. Cell plates were then cleaned with 100% IPA for 1 h and dried overnight at 55 °C in an oven.

#### Cell seeding and treatment

A549 adenocarcinoma cells (ATCC CCL-185) were maintained in DMEM/F12 media (Corning, 10-092-CV) supplemented with 10% FBS (VWR, 89510-186) and 1X Antibiotic-Antimycotic (Caisson Labs, 89510-186). Cells were detached from the cell plates using 0.25% trypsin-EDTA (Gibco, 25200-072). Approximately 1.65 × 10^4^ cells were seeded in each microwell. The cells were maintained on the cell plate for 2 or 3 days prior treatment.

3D printed chips with the 5-stage serial diluter were primed with TWEEN-80 (0.05% in deionized water) for 10 min to eliminate bubbles inside the device. Then both Fluid 1 (diluent, 3 μg/mL propidium iodide in DMEM/F12) and Fluid 2 (200 μg/mL digitonin + 3 μg/mL propidium iodide in DMEM/F12) were introduced into the device using two syringe pumps at 40 μL/min (Fluid 1) and 10 μL/min (Fluid 2) while agitating the inputs until there were no bubbles inside the chip. The syringe pump was then disconnected and the serial diluter was turned on and operated with a phase interval of 50 ms. After equilibration, the chip with cell-seeded wells was mounted and clamped on top of the serial diluter chip. The cells were treated for ~20 min, following which the cell plate was separated from the serial diluter. The cell plate was then immediately fixed using 1% paraformaldehyde in PBS (Thermo Fischer Scientific, 256956) for 15 min at 37 °C with mild agitation then incubated for 5 min with a PBS wash three times. The cell plate was then imaged using an Olympus IX73 microscope and ORCA-Flash 4.0 camera (Hamamatsu). A separate cell plate was used for the ethanol control. Each cell plate was soaked in 3 μg/mL propidium iodide diluted in 100% ethanol for 20 min at room temperature followed by an incubation of 5 min with a PBS wash three times.

#### Image processing and analysis

Fluorescence images were processed using Fiji (ImageJ 1.52p, National Institutes of Health USA) using the background removal function. Intensity signals were quantified as the area of positive cells using Fiji thresholding and area measurement. Visible light images were stitched using Adobe Photoshop CC 2018 (Adobe Systems Incorporated). Data were processed using Graphpad Prism Version 8.4.2 (Graphpad Software, LLC). Limits were set to the 100% ethanol as the 100% permeabilization response and 0 μg/mL digitonin as the baseline response.

#### μCT scans and light microscopy of the chips

Microchip wells and channels were filled with stabilized gram iodine (Difco, Detroit, MI) to provide contrast inside fluid channels. Chips were then scanned using a QuantumGX2 μCT scanner (PerkinElmer, Waltham, MA) at the following settings: 4 min High resolution, Al 0.5 mm + Cu 0.06 mm filter, 36 mm FOV, 90 kV, 88 μA. After initial scan images were reconstructed to a final voxel size of 25 μm and 9 μm. 3D images were then reconstructed as a Maximum Intensity Projection using Caliper Analyze 12.0 (AnalyzeDirect, Inc., Overland Park, KS). False coloring was achieved using Adobe Photoshop CC 2018. Photomicrographs of the chips are taken using a Canon EOS Rebel SL2 camera mounted on an Olympus BX51 microscope with UPlanFL N 4x objective. Image stitching basic light correction was done in Adobe Photoshop CC 2018.

## Supplementary information

Supplementary Information

Description of Additional Supplementary Files

Supplementary Movie 1

Supplementary Movie 2

Supplementary Movie 3

Supplementary Movie 4

Supplementary Movie 5

Supplementary Movie 6

Supplementary Movie 7

Supplementary Movie 8

## Data Availability

The data generated in this study have been deposited in the figshare database under accession code 10.6084/m9.figshare.14998332.v1^40^.
