# Supplementary material for: Spatially and optically tailored 3D printing for highly miniaturized and integrated microfluidics
Source: Nat Commun. 2021 Sep 17;12:5509. doi: 10.1038/s41467-021-25788-w (PMC8448845; doi:10.1038/s41467-021-25788-w)
Supplement: Supplementary file 1 — Supplementary Information [file 41467_2021_25788_MOESM1_ESM.pdf]

# Supplementary Information

# Spatially and optically tailored 3D printing for highly miniaturized and integrated microfluidics

Jose L. Sanchez Noriega[1], Nicholas A. Chartrand[2], Jonard Corpuz Valdoz[2], Collin G. Cribbs[2], Dallin A. Jacobs[2], Daniel Poulson[2], Matthew S. Viglione[1], Adam T. Woolley[2], Pam M. Van Ry[2], Kenneth A. Christensen[2], and Gregory P. Nordin[†1]

[1]Electrical and Computer Engineering Department, Brigham Young University, Provo, Utah, USA 84602

[2]Chemistry and Biochemistry Department, Brigham Young University, Provo, Utah, USA 84602

[†]Corresponding author: nordin@byu.edu

## Supplementary Note 1 Valves

The following videos show both membrane and squeeze valves in operation.

- **Supplementary Video 1**: membrane valve toggling between open and closed states; corresponds to the still images in Figs. 1(d-g) in the main text.
  - 20 pixel membrane valve (152 $\mu$m diameter).
  - Bottom view and side view in the left and right panels, respectively.
  - Actuation pressure: 25 psi
- **Supplementary Video 2**: membrane valve toggling between open and closed states; corresponds to the still images in Figs. 1(l-o) in the main text.
  - 6 pixel membrane valve (46 $\mu$m diameter).
  - Bottom view and side view in the left and right panels, respectively.
  - Actuation pressure: 9 psi
- **Supplementary Video 3**: squeeze valve toggling between open and closed states; corresponds to the still images in Figs. 1(s-v) in the main text.
  - 4 pixel valve (30 $\mu$m square active area).
  - Bottom view and side view in the left and right panels, respectively.
  - Actuation pressure: 40 psi left panel, 30 psi right panel.

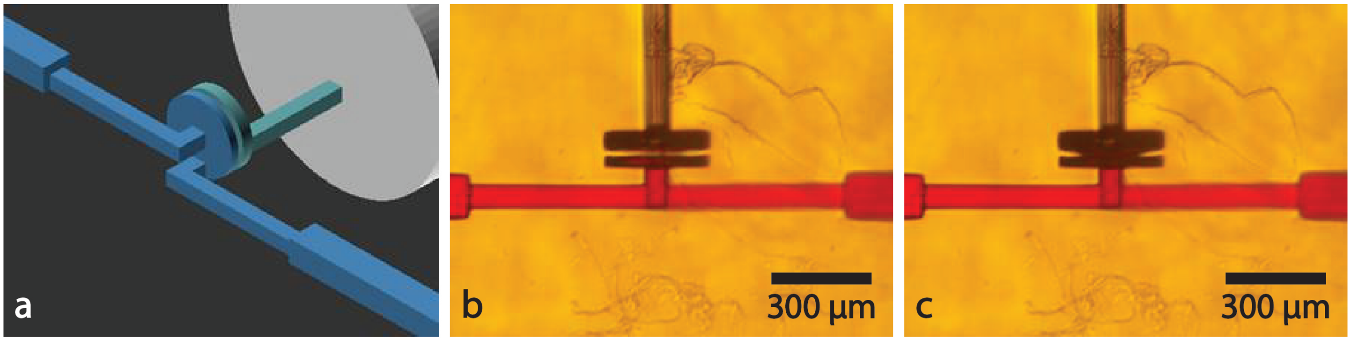


Supplementary Figure 1: **Vertically oriented membrane valve.** (a) Schematic, oblique view. (b-c) Microscope top-view photos under (b) open, and (c) closed (15 psi) states.

## Supplementary Note 2 Vertically oriented membrane valve

Supplementary Figure 1 shows a vertically oriented 3D printed membrane valve with a 300 $\mu$m diameter membrane. The vertical membrane is constructed as part of 30 successively 3D printed layers. This demonstrates the geometric versatility of the microfuidic designs that our 3D printer is able to create.

## Supplementary Note 3 Pumps

Supplementary Table 1 shows the 5 phase pump timing used in all membrane and squeeze valve based pumps. Supplementary Figure 2 illustrates the 5 pump phases for a pump comprised of three 4-pixel squeeze valves. The valves are labeled V1, DC, and V2, where the DC valve functions as a displacement chamber as explained in Sect. 4.3 of the main text. In Supplementary Figure 2, a squeeze valve is in a closed state when the central part of the valve looks bigger and has the same color as the horizontal (pneumatic control) lines, whereas it is in an open state when the central part appears smaller and is the same color as the vertical (fluid) lines, which are filled with an aqueous solution containing green food dye.

| | $t_0$ | $t_1$ | $t_2$ | $t_3$ | $t_4$ |
|---|---|---|---|---|---|
| $V_1$ | ● (red) | ● (green) | ● (red) | ● (red) | ● (red) |
| $DC$ | ● (red) | ● (green) | ● (green) | ● (green) | ● (red) |
| $V_2$ | ● (red) | ● (red) | ● (red) | ● (green) | ● (green) |

Supplementary Table 1: Pump timing logic. Red: valves closed; green: valves opened

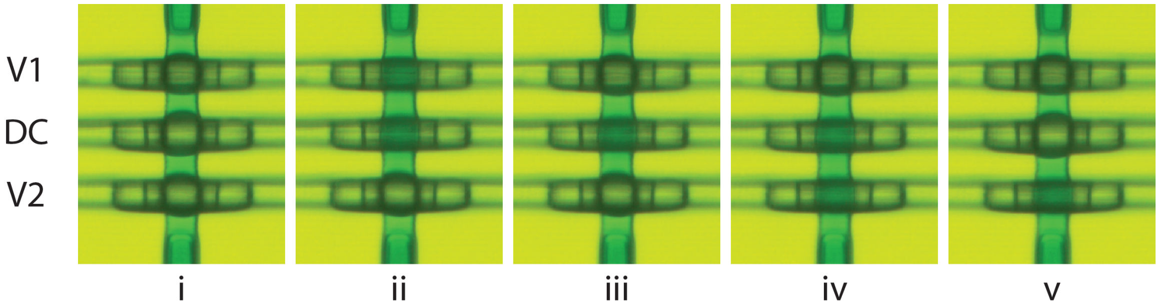


Supplementary Figure 2: **5-phase pumping cycle illustrated with a squeeze valve pump.** In (i) both valves and DC are closed, whereas in (ii) V1 and DC are open and V2 is closed. In (iii) V1 and V2 are closed and DC is open. In (iv) V1 remains closed and DC and V2 are open. In (v) V1 and DC are closed and V2 is open. At this point the pump sequence repeats, going back to (i).

The following videos show both membrane- and squeeze-valve-based pumps in operation.

- 20 pixel membrane valves and 20 pixel membrane DC
  - **Supplementary Video 4**: pumping dye through a vertical serpentine.
    * Side view
    * Actuation pressure: 25 psi
    * Phase interval: 50 ms
- 20 pixel membrane valves and 40 pixel membrane DC
  - **Supplementary Video 5**: pumping dye through a vertical serpentine.
    * Side view
    * Actuation pressure: 25 psi
    * Phase interval: 50 ms
- 4 pixel squeeze valves and DC
  - **Supplementary Video 6**: pumping dye through a vertical serpentine.
    * Side view
    * Vacuum applied
    * Actuation pressure: 35 psi
    * Phase interval: 100 ms

# Supplementary Note 4 Single stage 1:1 mixers

The 7-phase valve actuation schedule for the squeeze valve-based 1:1 mixer in Figs. 5(g) and 5(h) in the main text is shown in Supplementary Table 2.

| | $t_0$ | $t_1$ | $t_2$ | $t_3$ | $t_4$ | $t_5$ | $t_6$ |
|---|---|---|---|---|---|---|---|
| $L$ | red | green | red | red | red | red | red |
| $C$ | red | green | green | green | green | red | red |
| $R$ | red | red | red | red | green | green | red |
| $V_{out}$ | red | red | red | green | green | green | green |

Supplementary Table 2: Timing sequence for squeeze valve-based mixer. With reference to Fig. 5(g) in the main text, "L", "C", and "R" are the left, center, and right squeeze valves, respectively. Red: valves closed; green: valves open.

# Supplementary Note 5 Serial diluter

Supplementary Figure 3(a) shows a chip with a 10-stage, 2-fold serial diluter (top of chip) and a single-stage 1:1 mixer (bottom of chip), while Supplementary Figure 3(b) shows a microscope photo of two identical 10-stage serial diluter mixers on one chip. In the case of both chips, the structures were fabricated for characterization measurements. Around the border of each chip are cylindrical holes to which tubing is attached for fluid source and pneumatic control external connections.

Supplementary Figure 4 shows the relative output concentration of 3 different 10-stage serial diluter mixers, covering 3 orders of magnitude.

The following videos show both membrane- and squeeze-valve serial diluters in operation.

- Serial diluter with pumps comprised of 20 pixel membrane valves and 40 pixel membrane DCs.
  - **Supplementary Video 7**: dye fluid getting diluted serially. Outputs start with only water.

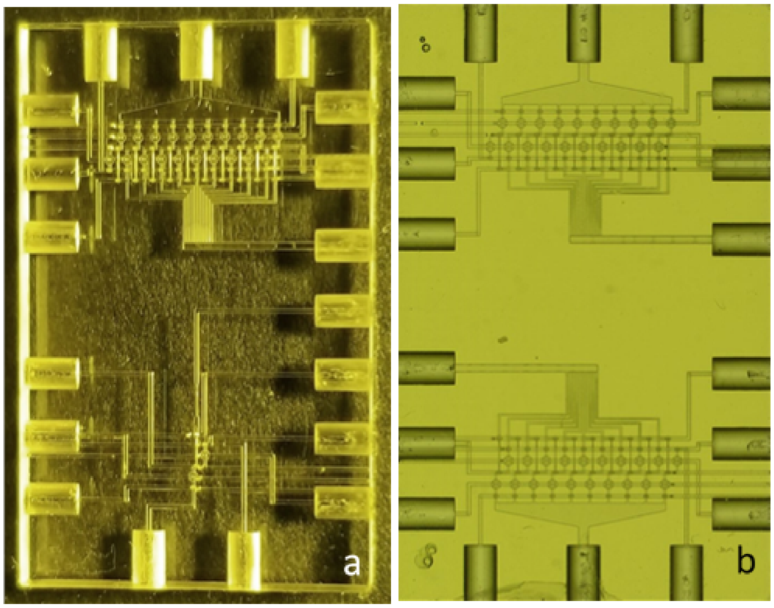


Supplementary Figure 3: **Serial dilution and mixer chips.** (a) Photo of a chip with a 10 stage serial dilution mixer and a single stage mixer. (b) Microscope photo of a chip with two 10 stage serial dilution mixers. Both chips are 19.5 mm (length) × 12.2 mm (width) × 2.8 mm (height).

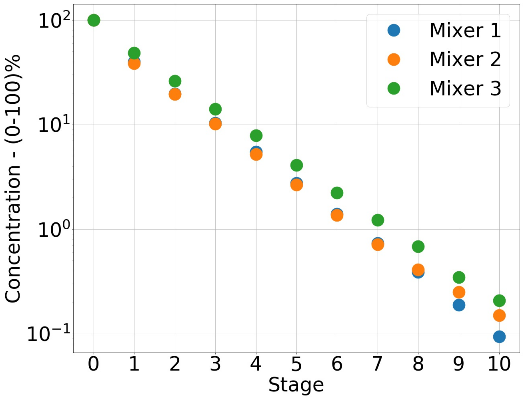


Supplementary Figure 4: **Measurement results.** Relative output concentrations of 3 different 10-stage serial diluter mixers.

    * Top view
    * Actuation pressure: 25 psi
    * Phase interval: 50 ms

- Serial diluter with pumps comprised of 4 pixel squeeze valves and DCs.
  - **Supplementary Video 8**: dye fluid getting diluted serially. Outputs start with only water.
    * Top view
    * Actuation pressure: 19 psi
    * Phase interval: 50 ms

## Supplementary Note 6 Digitonin assay

Supplementary Figure 5 provides additional detail for the diluter device integration with the microfluidic cell plate. Supplementary Figure 6 shows representative differential interference contrast (DIC) optical microscope images and corresponding fluorescence images for the digitonin assay.

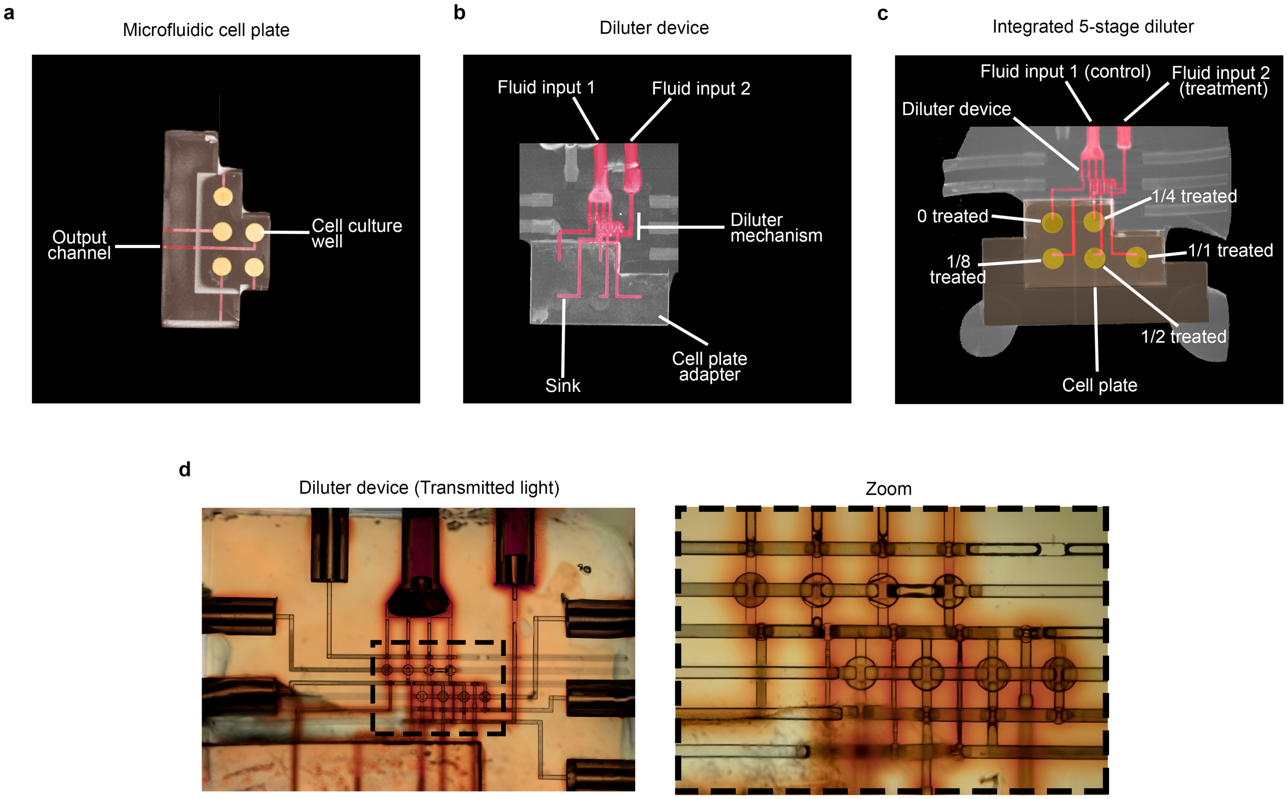


Supplementary Figure 5: **Digitonin assay device.** (a-c) Images of the iodine-perfused devices imaged using $\mu$CT scan. (a) The microfluidic cell plate treated with $O_2$ plasma for cell adherence. This features cell culture microwells (yellow circles, 1.78 mm in diameter) and output channels (red). (b) Diluter device with two fluid inputs which feed the diluter mechanism. The diluted fluids are pumped to the sink, which contains 5 outlet channels. (c) Integrated 5-stage diluter showing the microfluidic cell plate (brown) fitting onto the diluter device (grey). In this paper, the fluid inputs were perfused with control (diluent) and treatment fluids. These fluids pass through the diluter mechanism which pumps the diluted fluids to the sink which directly fills the cell culture wells with the appropriate dilutions of the treatment, which in this paper, is digitonin. (d) The same device was imaged through transmitted light microscopy with image stitching. Zoomed image shows the details of the diluter mechanism piping system.

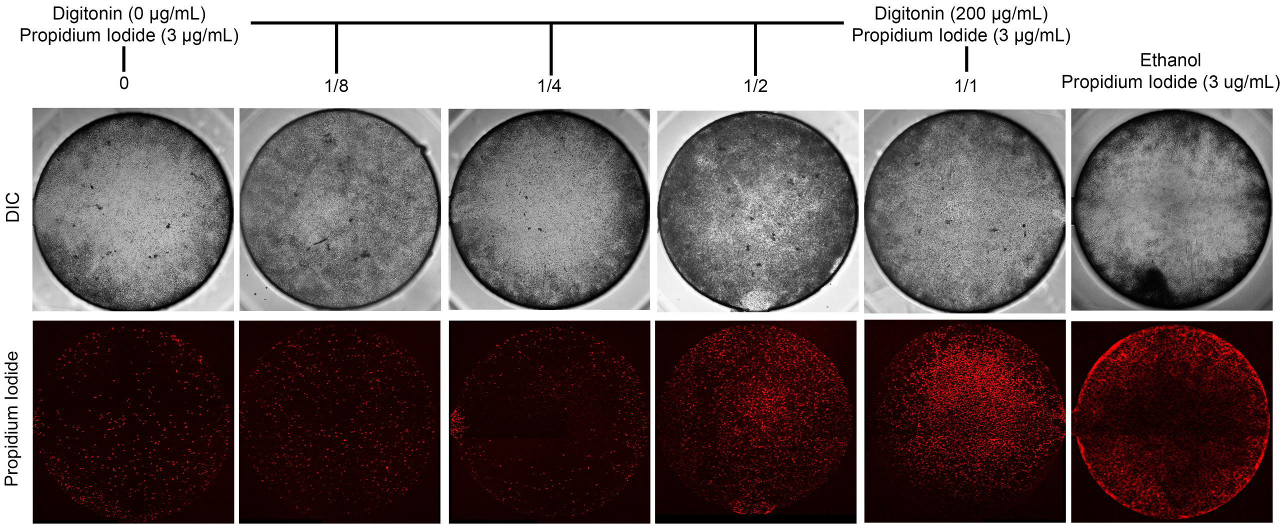


Supplementary Figure 6: **Representative images for the digitonin-induced permeabilization of A549 cells.** The top row shows the dilution profile by the device. The middle row shows the virtually equal cell coverage between cell culture wells as imaged through DIC. The bottom row shows the propidium iodide signal. Each microwell has a diameter of 1.78 mm.

# Supplementary Note 7 Parallel Device Printing

As shown in Supplementary Figure 7, many devices can be 3D printed simultaneously in a single print run if individual devices are small enough. In this example we have chosen a simple pump design with 40 pixel (304 $\mu$m) diameter valves and displacement chamber. Supplementary Figure 7(a) shows 117 pump chips, each $0.88 \times 2.1 \times 1.0$ mm$^3$, fabricated in parallel. Supplementary Figure 7(b) shows a close-up of several chips before singulation. For each pump chip, the required 2 fluid connections and 3 pneumatic connections are implemented as vertical chip-to-chip interconnects such that a single device chip can be clamped to an interface chip to form leak-free connections between the two. The interface chip, Supplementary Figure 7(d), implements a chip-to-world interface which consists of separate PTFE tubes glued into the interface chip. The interface chip also contains a serpentine channel. Supplementary Figure 7(e) shows a close-up microscope image of the serpentine channel (in focus) and the pump chip (out of focus because it is below the microscope plane of focus clamped to the bottom surface of the interface chip). The pumps and interface chip operated as designed.

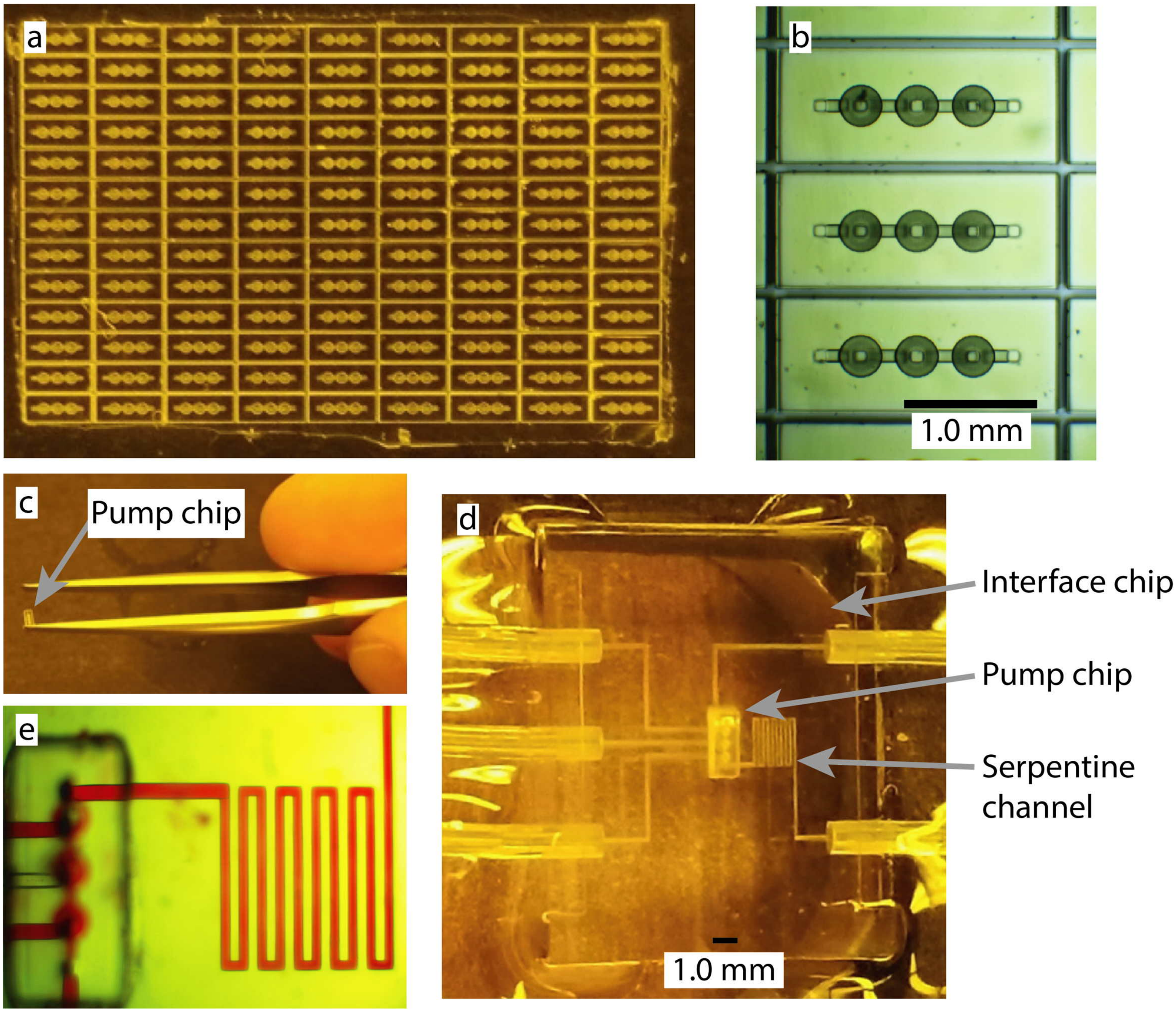


Supplementary Figure 7: **Parallel printing of 117 pumps in one print run.** (a) All 117 pumps just after 3D printing. (b) Close-up microscope image of several pumps before singulation. (c) Single pump chip on tweezers to show scale. (d) Pump chip integrated with interface chip. (e) Fluid channels filled with red aqueous solution.
