# Supplementary material for: Spatially and optically tailored 3D printing for highly miniaturized and integrated microfluidics
Source: Nat Commun. 2021 Sep 17;12:5509. doi: 10.1038/s41467-021-25788-w (PMC8448845; doi:10.1038/s41467-021-25788-w)
Supplement: Supplementary file 2 — Description of Additional Supplementary Files [file 41467_2021_25788_MOESM2_ESM.docx]

**Description of Additional Supplementary Files**

**File Name:** Supplementary Video 1

**Description:** 20 pixel membrane valve (152 μm) toggling between open and closed states with 25 psi actuation pressure.

**File Name:** Supplementary Video 2

**Description:** 6 pixel membrane valve (46 μm) toggling between open and closed states with 9 psi actuation pressure.

**File Name:** Supplementary Video 3

**Description:** 4 pixel squeeze valve (30 μm) toggling between open and closed states with 40 and 30 psi actuation pressure, respectively, for the left and right panels.

**File Name:** Supplementary Video 4

**Description:** Pump operation – 20 pixel (152 μm) membrane valves and displacement chamber with 25 psi actuation pressure and 50 ms phase interval.

**File Name:** Supplementary Video 5

**Description:** Pump operation – 20 pixel (152 μm) membrane valves and 40 pixel (304 μm) displacement chamber with 25 psi actuation pressure and 50 ms phase interval.

**File Name:** Supplementary Video 6

**Description:** Pump operation – 4 pixel (30 μm) squeeze valves and displacement chamber with 35 psi actuation pressure and 100 ms phase interval.

**File Name:** Supplementary Video 7

**Description:** Serial diluter operation – membrane valve-based version with 25 psi actuation pressure and 50 ms phase interval.

**File Name:** Supplementary Video 8

**Description:** Serial diluter operation – squeeze valve-based version with 19 psi actuation pressure and 50 ms phase interval.
